# *Cirsium japonicum* var. *maackii* and apigenin block Hif‐2α‐induced osteoarthritic cartilage destruction

**DOI:** 10.1111/jcmm.14418

**Published:** 2019-05-31

**Authors:** Chanmi Cho, Li‐Jung Kang, Dain Jang, Jimin Jeon, Hyemi Lee, Sangil Choi, Seong Jae Han, Eunjeong Oh, Jiho Nam, Chun Sung Kim, Eunkuk Park, Seon‐Yong Jeong, Chan Hum Park, Yu Su Shin, Seong‐il Eyun, Siyoung Yang

**Affiliations:** ^1^ Department of Biomedical Sciences Ajou University Graduate School of Medicine Suwon Korea; ^2^ Department of Pharmacology Ajou University School of Medicine Suwon Korea; ^3^ CIRNO, Sungkyunkwan University Suwon Korea; ^4^ Department of Oral Biochemistry, College of Dentistry Chosun University Gwangju Korea; ^5^ Department of Medical Genetics Ajou University School of Medicine Suwon Korea; ^6^ Department of Medicinal Crop Research, National Institute of Horticultural and Herbal Science Rural Development Administration Eumseong Korea; ^7^ Department of Life Science Chung‐Ang University Seoul Korea

**Keywords:** apigenin, *Cirsium japonicum* var. *maackii*, Cox‐2, Hif‐2α, Mmp, osteoarthritis

## Abstract

Although Hif‐2α is a master regulator of catabolic factor expression in osteoarthritis development, Hif‐2α inhibitors remain undeveloped. The aim of this study was to determine whether *Cirsium japonicum* var. *maackii* (CJM) extract and one of its constituents, apigenin, could attenuate the Hif‐2α‐induced cartilage destruction implicated in osteoarthritis progression. In vitro and in vivo studies demonstrated that CJM reduced the IL‐1β‐, IL‐6, IL‐17‐ and TNF‐α‐induced up‐regulation of MMP3, MMP13, ADAMTS4, ADAMTS5 and COX‐2 and blocked osteoarthritis development in a destabilization of the medial meniscus mouse model. Activation of Hif‐2α, which directly up‐regulates MMP3, MMP13, ADAMTS4, IL‐6 and COX‐2 expression, is inhibited by CJM extract. Although cirsimarin, cirsimaritin and apigenin are components of CJM and can reduce inflammation, only apigenin effectively reduced Hif‐2α expression and inhibited Hif‐2α‐induced MMP3, MMP13, ADAMTS4, IL‐6 and COX‐2 expression in articular chondrocytes. IL‐1β induction of JNK phosphorylation and IκB degradation, representing a critical pathway for Hif‐2α expression, was completely blocked by apigenin in a concentration‐dependent manner. Collectively, these effects indicate that CJM and one of its most potent constituents, apigenin, can lead to the development of therapeutic agents for blocking osteoarthritis development as novel Hif‐2α inhibitors.

## 1 INTRODUCTION

Cartilage destruction and joint inflammation are the main causes of osteoarthritis (OA), which reduces patient's quality of life.^1^, ^2^ The molecular mechanisms of inflammation and cartilage destruction involve the induction of catabolic factors, such as matrix metalloproteinases (MMPs), a disintegrin and metalloproteinases with thrombospondin motifs (ADAMTS) and cyclooxygenase 2 (COX‐2), in articular chondrocytes.^3^ Pro‐inflammatory cytokines (eg, interleukin (IL)‐1β, IL‐6, IL‐17 and tumour necrosis factor (TNF)‐α) and mechanical stress can induce MMPs, ADAMTS and COX‐2 expression and accelerate cartilage destruction and OA development.^4^, ^5^, ^6^

Among the numerous MMP isotypes, MMP3 and MMP13 play major roles in cartilage matrix degradation.^7^, ^8^ MMP3 and MMP13 possess aggrecanase and collagenase activities, respectively, and they degrade aggrecan, type II collagen and other extracellular matrix (ECM) components.^9^, ^10^ Furthermore, ADAMTS4 and ADAMTS5 cleave aggrecan in vivo and are responsible for the up‐regulated aggrecanase activity observed in OA development.^11^ Although COX‐2 is mainly involved in joint inflammation, this further stimulates ECM degradation by activating MMPs and ADAMTS during OA progression.^11^, ^12^, ^13^ Thus, ECM components are depleted during OA development by increased expression of COX‐2, MMPs and ADAMTS.

The hypoxia‐inducible factor 2α (Hif‐2α) transcription factor is a known master regulator of OA pathogenesis and enhances expression of MMP3, MMP13, COX‐2 and other catabolic factors via specific binding to hypoxia‐responsive elements (‐CGTG‐) in their promoter regions.^14^ Moreover, pro‐inflammatory cytokines (eg, IL‐1β, IL‐6 and TNF‐α) can up‐regulate Hif‐2α expression in articular chondrocytes mainly through nuclear factor (NF)‐κB and c‐Jun N‐terminal kinase (JNK) signalling pathways.^6^, ^14^ Although the regulation of catabolic factors by Hif‐2α inhibition is a good therapeutic strategy to attenuate OA progression, a safe natural substance or a single compound that can inhibit Hif‐2α is still lacking.

*Cirsium japonicum* var. *maackii* (CJM), a member of the composite family, is a safe perennial herb that has been used as a traditional antihaemorrhagic, antihypertensive, anti‐hepatitis and uretic medicine.^15^, ^16^, ^17^ Several compounds, including cirsimarin, cirsimaritin and apigenin, have been identified in CJM and these compounds are important for the pharmaceutical activities.^18^ Although several studies have been conducted on the effects of cirsimarin, cirsimaritin and apigenin on cancer development, hepatoprotection, inflammation and diabetes,^19^, ^20^, ^21^, ^22^ the function of CJM and its isolated constituents on attenuating OA progression is still unknown.

Accordingly, the purpose of this study was to investigate the effect of CJM and one of its components, apigenin, in regulating MMP3, MMP13, ADAMTS4, ADAMTS5 and COX‐2 expression and protecting OA development, using both in vitro and in vivo analyses. Furthermore, these experiments will focus on Hif‐2α regulation by CJM and apigenin and will determine whether they act as Hif‐2α inhibitors and have therapeutic potential in OA treatment.

## 2 MATERIALS AND METHODS

### 2.1 Reagents and treatment

Samples of the dried aerial part of *Cirsium japonicum* var. *maackii* (CJM) were obtained commercially from Imsil Herbal Medicine (Imsil‐gun, Jeollabuk‐do, Korea). The dried samples were mixed with 30% ethanol and the mixture was refluxed at 65°C for 3 hours. Total CJM extract was obtained as the filtrate after vacuum filtration at 25°C. Apigenin, cirsimarin and cirsimaritin were purchased from Sigma‐Aldrich (St. Louis, MO, USA). Pro‐inflammatory cytokines (IL‐1β, IL‐6, IL‐17 and TNF‐α) were purchased from GenScript (Piscataway, NJ, USA). Mouse articular chondrocytes were treated with IL‐1β (1 ng/mL), IL‐6 (100 ng/mL), IL‐17 (10 ng/mL) or TNF‐α (50 ng/mL) and co‐treated with CJM extract (10, 50 or 100 μg/mL), apigenin (10, 25 or 50 μmol/L), cirsimarin (10, 25 or 50 μmol/L) or cirsimaritin (10, 25 or 50 μmol/L) for 24 h before they were harvested.

### 2.2 HPLC analysis of cirsimarin, cirsimaritin and apigenin

Quantitative analysis of cirsimarin, cirsimaritin and apigenin in the CJM extract was performed with a Waters Breeze System HPLC (Waters Co., Milford, MA, USA) equipped with a 250 mm × 4.6 mm (*df* = 5 μm) C18 INNO column and a UV/VIS detector (set at 270 nm). The concentrations of cirsimaritin, cirsimarin and apigenin in the extract were determined from corresponding calibration curves. Figure 4A shows representative HPLC spectra of the CJM extract and standard.

### 2.3 Culture of mouse articular chondrocytes and chondrocyte viability assay

Articular chondrocytes were isolated from femoral condyles and tibial plateaus of post‐natal day 5 mice. Cartilage tissues were digested with 0.2% collagenase type II, as previously described.^23^ For the chondrocyte viability assay, the chondrocytes were seeded in a 96‐well dish (9 × 10^3 ^cells/well) for 48 hours prior to treatments. CJM or apigenin was added at various concentrations and incubated for 24 hours in DMEM without foetal bovine serum. Cytotoxicity was assessed with the EZ‐CyTox Cell Viability Assay kit (Dogen, Seoul, South Korea) and an LDH Colorimetric assay kit (BioVision Inc, Milpitas, CA, USA) following the manufacturer's manual. Briefly, 2‐(4‐iodophenyl)‐3‐(4‐nitrophenyl)‐5‐(2,4‐disulfophenyl)‐2H‐tetrazolium (WST‐1) solution mixed with serum‐free DMEM (1:100, v/v) was added to the cultured cells and incubated for 3 hours. To analyse LDH activity, cell viability was normalized to that of untreated samples (100% viability) and samples treated with Triton X‐100 (0% viability). Assays were performed on the supernatants of chondrocytes at time points of 36 hours post‐treatment of CJM and apigenin at various concentrations. % cytotoxicity was calculated by the formula (Sample LDH − Negative Control)/(Max LDH − Negative Control) × 100. The absorbance was measured using a microplate reader (VICTOR X3; PerkinElmer, Waltham, MA) at 450 and 595 nm for WST‐1 and LDH assays respectively.

### 2.4 Reverse transcription‐polymerase chain reaction and quantitative RT‐PCR

Total RNA was isolated from articular chondrocytes using TRIzol (Molecular Research Center Inc, Cincinnati, OH, USA). Total RNA was reverse transcribed and the resulting cDNA was amplified by PCR (iTaq, Intron Biotechnology, Gyeonggi‐do, South Korea). The PCR primers are summarized in Table S1. The transcript levels of target genes were quantified by quantitative reverse transcription‐polymerase chain reaction (qRT‐PCR) using SYBR^®^ premix Ex Taq (TaKaRa Bio, Shiga, Japan). For each target gene, the transcript level was normalized to that of *Gapdh* and expressed as a fold change relative to the indicated control.

### 2.5 Western blotting and immunohistochemistry

Total proteins were extracted with lysis buffer (150 mmol/L NaCl, 1% NP‐40, 50 mmol/L Tris, 0.2% sodium dodecyl sulphate and 5 mmol/L NaF) supplemented with a protease inhibitor and phosphatase inhibitor cocktail (Roche, Madison, WI, USA). MMP3 and MMP13 were detected after trichloroacetic acid precipitation as described previously.^23^ Each protein was visualized using the SuperSignal West Dura kit (Thermo Scientific, Waltham, MA, USA); total extracellular signal‐regulated kinase (ERK) was used as a loading control. Western blot analysis was performed to detect protein levels using the following antibodies: mouse anti‐Hif‐2α (sc‐13596; Santa Cruz Biotechnology, Dallas, TX, USA), mouse anti‐Mmp3 (ab52915; Abcam, Cambridge, UK), mouse anti‐Mmp13 (ab51072; Abcam), goat anti‐COX‐2 (sc‐1745; Santa Cruz), mouse anti‐Erk1/2 (610408; Becton Dickinson, NJ, USA), mouse anti‐pErk1/2 (9101; Cell Signaling Technology, Boston, MA, USA) and mouse anti‐IκB (9242; Cell Signaling Technology). Anti‐Hif‐2α antibody (ab8365; Abcam) was used for immunostaining as previously described.^14^

### 2.6 Experimental OA models and oral gavage

All animal experiments were approved by the Animal Care and Use Committee of the University of Ajou. For the destabilization of the medial meniscus (DMM)‐induced OA model, 12‐week‐old male C57BL/6 mice were subjected to DMM surgery using a previously described protocol.^24^ Mouse knee joints were processed for histological analysis 10 weeks after surgery. Experimental OA was also induced in 12‐week‐old male mice by intraarticular (IA) injection (once weekly for 3 weeks) of Hif‐2α‐expressing adenovirus (Ad‐Hif‐2α; 1 × 10^9^ plaque‐forming units in a total volume of 10 µL); IA injection of empty adenovirus (Ad‐C) was used as a control. The DMM‐induced and Ad‐Hif‐2α‐induced OA models received CJM by gavage every other day for 10 weeks and 3 weeks, respectively, and were killed after completion of the gavage feeding.

### 2.7 Evaluation of cartilage destruction

Cartilage destruction was assessed as previously described.^25^ Briefly, mouse knee joints were fixed in 4% paraformaldehyde, decalcified with 0.5 mol/L EDTA (pH 8.0) for 2 weeks and embedded in paraffin. The paraffin blocks were sectioned at a thickness of 5 μm and were serially sectioned at 40‐μm intervals. The sections were deparaffinized in xylene and hydrated with a graded ethanol series. Cartilage destruction was detected by Safranin O staining and scored using the Osteoarthritis Research Society International (OARSI) grading system.

### 2.8 Cartilage explants and 1,9‐dimethylmethylene blue assay

Mouse femoral head cartilage was dissected from 3‐week‐old C57BL/6 mice and incubated in DMEM (Gibco‐BRL) containing CJM or apigenin with or without IL‐1β (10 ng/mL) for 72 hours without changing the medium, as described previously.^23^ The conditioned medium from each well was collected to quantify aggrecan‐release content. The culture medium was mixed with 1,9‐dimethylmethylene blue (DMMB) solution (40 mmol/L NaCl, 40 mmol/L Glycine, 46 μmol/L DMMB) at 1:10 and the absorbance was measured at 525 nm within 10 minutes using a microplate reader. Bovine cartilage chondroitin sulphate was used for the standard curve of sulphated glycosaminoglycan (sGAG) content. For mouse knee joints, cartilage explants were obtained and cultured in DMEM (Gibco‐BRL) containing CJM or apigenin with or without IL‐1β (1 ng/mL) for 72 hours. The accumulation of sGAG was assessed by Alcian blue staining (1 volume of 0.3% Alcian blue 8GX in 70% ethanol, 1 volume of 100% acetic acid and 18 volumes of 100% ethanol), as described previously.^26^

### 2.9 Reporter gene assay

The Mmp3, Mmp13, Cox‐2 and Adamts4 reporter gene constructs were co‐transfected with or without Hif‐2α expression vector into mouse articular chondrocytes using LipofectAMINE Plus (Invitrogen, Carlsbad, CA, USA) as described previously.^14^ The transfected cells were cultured in complete medium for 24 hours. Luciferase activity was determined using an assay kit (Promega, Madison, WI, USA) and subsequently normalized to β‐galactosidase activity.

### 2.10 Statistical analysis

All experiments were performed independently at least three times. Statistical comparisons of two independent groups were made using two‐tailed independent *t* tests. Multiple comparisons were made using ANOVA with a post‐hoc Bonferroni test. Data based on ordinal grading systems, such as OARSI grade and subchondral bone thickness, were analysed using a non‐parametric Mann–Whitney *U* test. A *P*‐value of 0.05 was considered significant.

## 3 RESULTS

### 3.1 CJM inhibits Mmp3, Mmp13 and Cox‐2 expression under in vitro conditions mimicking OA

We first determined whether CJM demonstrates cytotoxicity towards chondrocytes. Treatment of the mouse articular chondrocytes with different concentrations of CJM for 36 hours resulted in no observable cytotoxicity as determined by WST‐1 assay, LDH assay and CJM‐induced catabolic factor expression (Figure S1).

**Figure 1:**
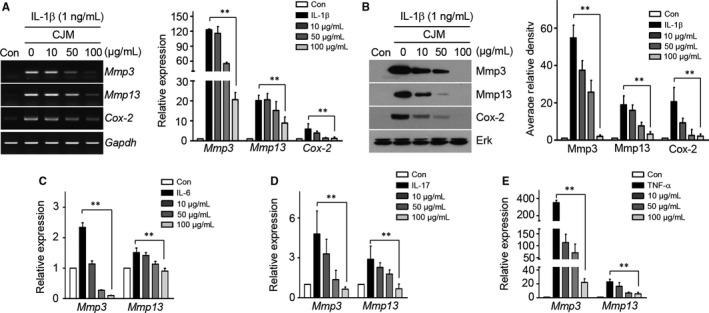
*Cirsium japonicum* var. *maackii* inhibits IL‐1β‐, IL‐6‐, IL‐17‐ and TNF‐α‐induced Mmp3, Mmp13 and Cox‐2 expression in articular chondrocytes. A and B, Chondrocytes treated with IL‐1β (1 ng/mL) were treated with or without various concentrations of *Cirsium japonicum* var. maackii extract for 24 h (n = 5). The expression of Mmp3, Mmp13 and Cox‐2 was determined by RT‐PCR (A, left), qRT‐PCR (A, right), Western blot (B, left) and densitometry (B, right). *Gapdh* and ERK were used as loading controls for PCR and Western blotting respectively. Chondrocytes treated with IL‐6 (100 ng/mL) (C), IL‐17 (10 ng/mL) (D) and TNF‐α (50 ng/mL) (E) were co‐treated with *Cirsium japonicum* var. maackii extract for 24 h at the indicated concentrations. The expression of Mmp3, Mmp13 and Cox‐2 was determined by qRT‐PCR. Data were analysed using two‐tailed *t* tests. Values represent the means ± SEM; **P < 0.005

Cartilage degradation and inflammation lead to OA development and progression.^1^, ^2^ We postulated that CJM could block cartilage degradation and inflammation via regulation of Mmp3, Mmp13, Adamts4, Adamts5 and Cox‐2 expression. To test the effect of CJM on mouse articular chondrocytes, the expression levels of Mmp3, Mmp13, Adamts4, Adamts5 and Cox‐2 were assessed by biochemical analysis. As shown in Figure 1A and 1 and Figure S2A, the expression levels of IL‐1β‐induced Mmp3, Mmp13, Adamts4, Adamts5 and Cox‐2 were gradually down‐regulated by CJM in a concentration‐dependent manner.

Although IL‐1β is an important OA‐related pro‐inflammatory cytokine, other pro‐inflammatory cytokines (eg, IL‐6, IL‐17 and TNF‐α) have recently been found to play important roles in OA development.^5^, ^6^, ^14^ Notably, CJM reduced IL‐6‐, IL‐17‐ or TNF‐α‐stimulated Mmp3, Mmp13, Adamts4 and Adamts5 expression (Figure 1C–E, Figure S2C–E). These results indicate that CJM can alleviate cartilage degradation and inflammation under in vitro OA‐mimetic conditions and, therefore, potentially protect cartilage from destruction during OA progression and development.

### 3.2 Oral administration of CJM protects against cartilage destruction in the DMM‐induced OA model and ex vivo organ culture system

**Figure 2:**
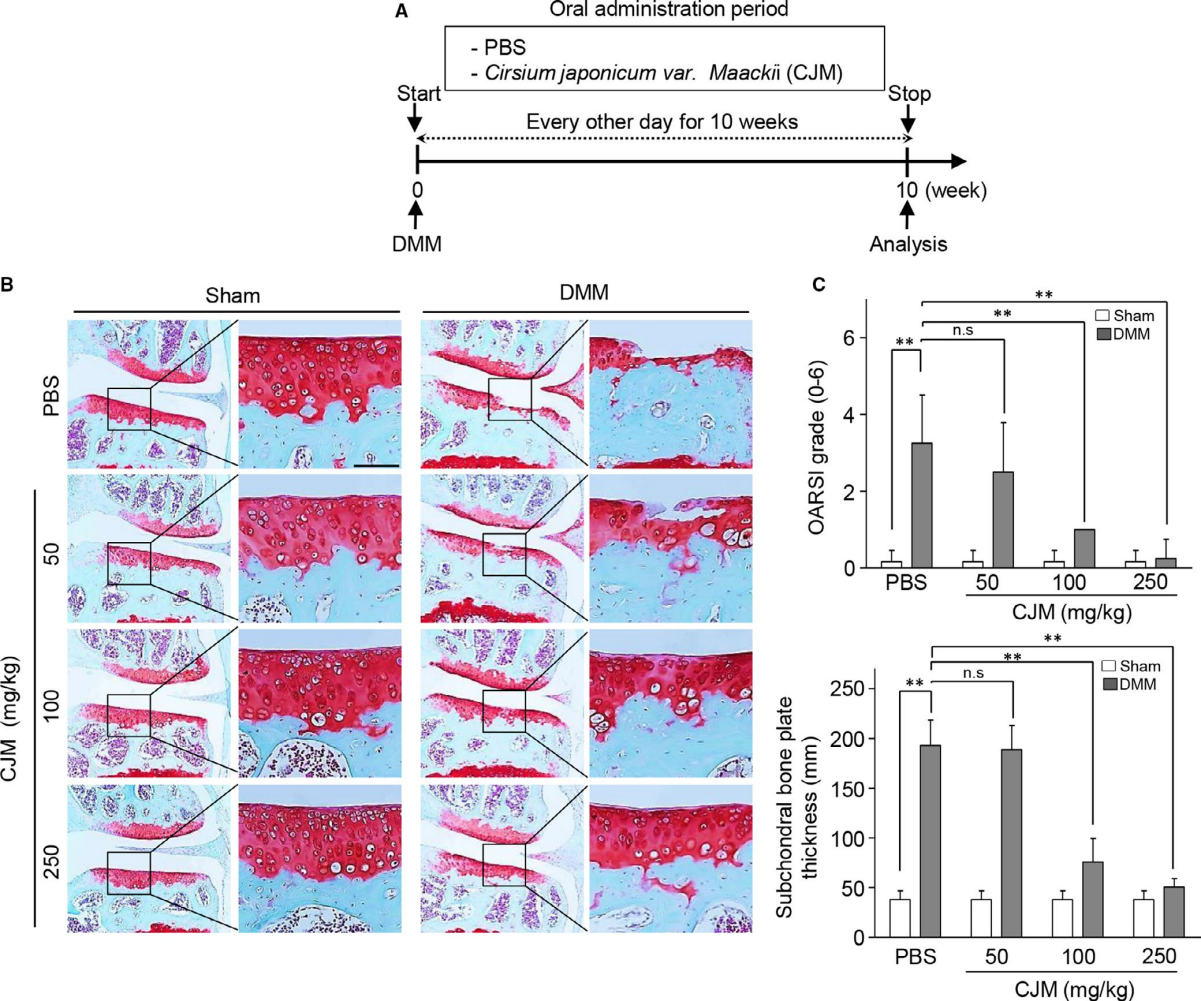
Oral administration of *Cirsium japonicum* var. *maackii* protects against cartilage destruction in OA development. A, Experimental scheme for analysis of the DMM‐induced OA model. B, Mice with DMM were treated three times a week with *Cirsium japonicum* var. *maackii* extract or PBS via oral gavage for 10 weeks. Cartilage destruction was detected by Safranin O staining. C, Cartilage destruction was quantified by OARSI scores (upper panel) and subchondral bone thickness (lower panel) at 10 weeks following surgery (n = 10). Scale bar: 50 µm. Data were analysed using one‐way ANOVA with Bonferroni's test or a non‐parametric Mann‐Whitney *U* test. Values represent the means ± SEM; **P < 0.005

To evaluate the role of CJM under in vivo conditions, we tested whether administration of CJM protects against osteoarthritic cartilage destruction in the DMM‐induced OA model. The mice were orally administered either PBS or PBS containing CJM every other day for 10 weeks, starting at DMM (Figure 2A). Compared to that of the PBS control group, oral administration of CJM resulted in dramatic protection against OA development (Figure 2B) as indicated by significantly lower OARSI scores and subchondral bone plate thickness (Figure 2C). Moreover, CJM restored the accumulation of extracellular sulphated proteoglycans and inhibited aggrecan release in IL‐1β‐stimulated cartilage explants (Figure S3A and B). Therefore, these results indicate that CJM may activate cartilage regeneration to facilitate OA development.

### 3.3 Hif‐2α expression is regulated by CJM in mouse articular chondrocytes

**Figure 3:**
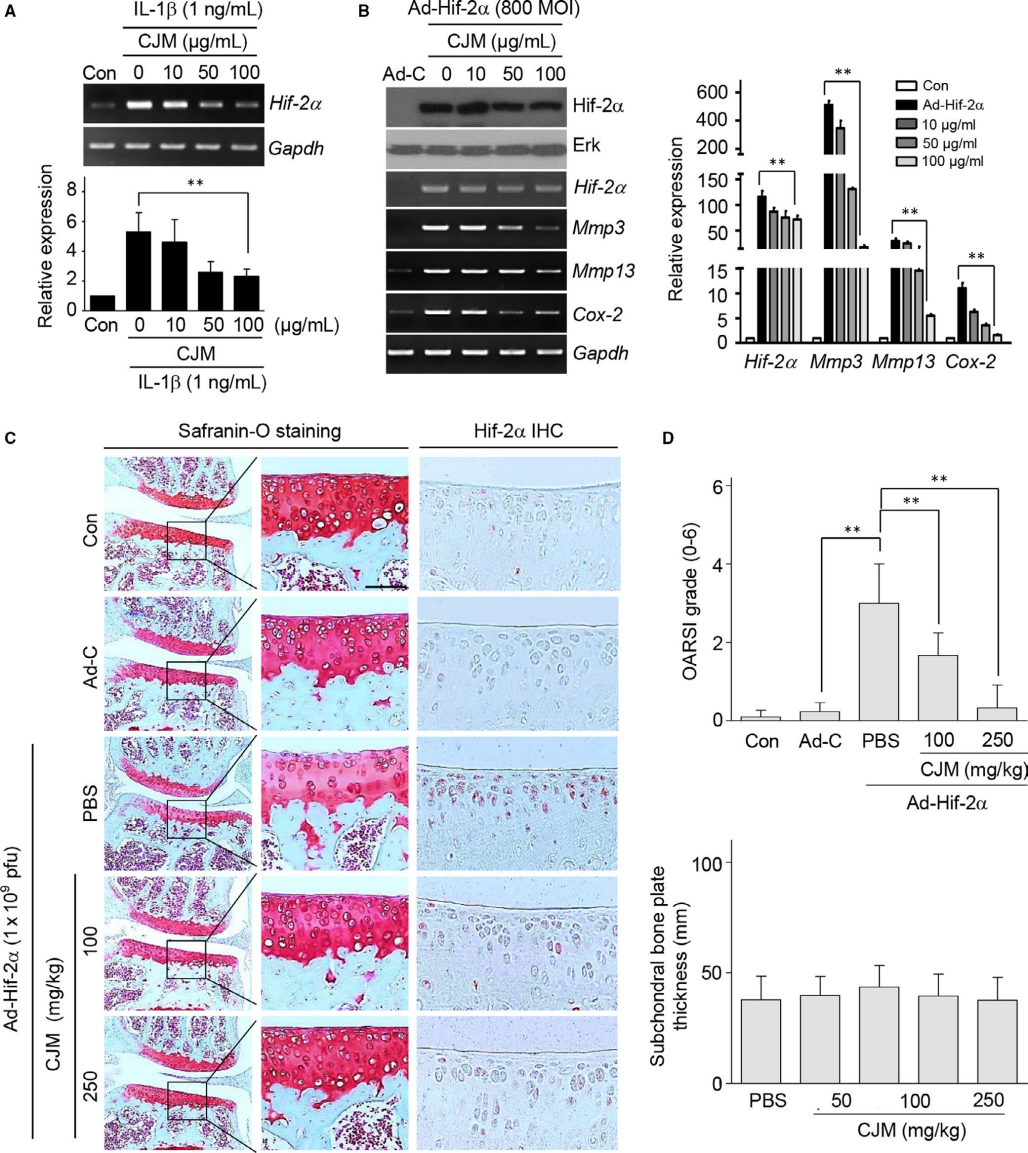
*Cirsium japonicum* var. *maackii* reduces Hif‐2α overexpression‐induced Mmp3, Mmp13 and Cox‐2 expression and attenuates Hif‐2α overexpression‐induced cartilage destruction. A, Chondrocytes were either untreated or treated with the indicated amount of *Cirsium japonicum* var. *maackii* extract and exposed to IL‐1β for 24 h (n = 5). Hif‐2α expression was analysed by RT‐PCR (upper panel) and qRT‐PCR (lower panel). B, Chondrocytes were transduced with Ad‐C or Ad‐Hif‐2α (800 MOI) and then treated with the indicated concentrations of *Cirsium japonicum* var. *maackii* for 24 h (n = 5). Western blot, RT‐PCR (left panel) and qRT‐PCR (right panel) are shown. Representative RT‐PCR gels are presented in A from more than five independent experiments using different sets of primary chondrocyte cultures. C and D, Mice were IA‐injected with Ad‐C or Ad‐Hif‐2α (1 × 10^9^ PFU) and then orally administered *Cirsium japonicum* var. *maackii* extract (n = 10). Representative images of cartilage sections stained with Safranin O (C), OARSI grades (D, upper panel) and subchondral bone plate thickness (D, lower panel) are shown. Scale bar: 50 µm. Data were analysed using two‐tailed *t*‐tests (B) and one‐way ANOVA with Bonferroni's test (D). Values represent the means ± SEM; **P < 0.005

Hif‐2α plays a central role in catabolic factor expression and works as a master regulator for inducing OA development.^6^, ^14^, ^27^ The level of Hif‐2α was increased by in vitro conditions that mimicked OA and Hif‐2α‐overexpressing chondrocytes demonstrated up‐regulated Mmp3, Mmp13, Adamts4 and COX‐2 expression (Figure S4). Therefore, we determined whether Hif‐2α levels or Hif‐2α‐induced Mmp3, Mmp13, Adamts4 and Cox‐2 expressions were regulated by CJM in mouse articular chondrocytes. Treatment of chondrocytes with IL‐1β up‐regulated Hif‐2α and this up–regulation was attenuated by CJM in a concentration‐dependent manner as determined by RT‐PCR and qRT‐PCR (Figure 3A). Furthermore, Mmp3, Mmp13, Adamts4 and Cox‐2 induction by Hif‐2α overexpression was reduced by CJM treatment in cultured mouse articular chondrocytes (Figure 3B, Figure S5A).

To assess the protective effect of CJM in Hif‐2α‐induced OA development, cartilage destruction and Hif‐2α expression were detected by Safranin O staining and immunohistochemistry respectively. As shown in Figure 3C and 3, although IA injection of Ad‐Hif‐2α promoted cartilage destruction in a mouse model, oral administration of CJM ameliorated this cartilage destruction. Moreover, Hif‐2α expression in the cartilage tissue of mice after IA injection with Ad‐Hif‐2α was dramatically reduced by CJM administration.

These results clearly indicate that Hif‐2α overexpression‐induced catabolic factors and cartilage destruction were regulated by CJM through Hif‐2α suppression in both cultured articular chondrocytes and mice.

### 3.4 Apigenin from CJM is a key component for regulating Hif‐2α expression

**Figure 4:**
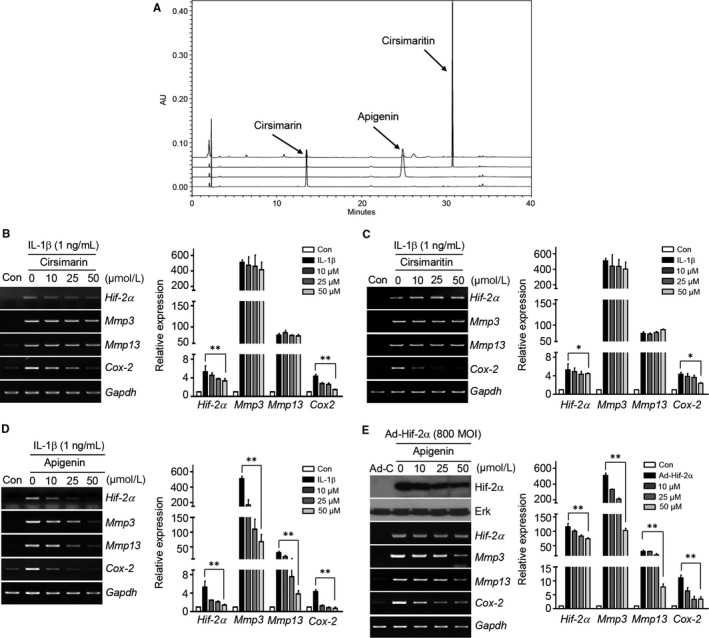
Apigenin regulates catabolic factor expression via Hif‐2α regulation. A, HPLC spectra of cirsimarin, cirsimaritin and apigenin in *Cirsium japonicum* var. *maackii* and the standard compounds. B‐D, Chondrocytes were either untreated or treated with the indicated amount of cirsimarin (B), cirsimaritin (C) or apigenin (D) and exposed to IL‐1β for 24 h (n = 5). Expression of Hif‐2α, Mmp3, Mmp13 and Cox‐2 was analysed by RT‐PCR (left panel) and qRT‐PCR (right panel). E, Chondrocytes were transduced with Ad‐C or Ad‐Hif‐2α (800 MOI) and then treated with the indicated concentrations of apigenin for 24 h. Western blot, RT‐PCR (left panel) and qRT‐PCR (right panel) are shown (n = 5). Representative RT‐PCR gels are presented in A‐D from more than five independent experiments using different sets of primary chondrocyte cultures. Data were analysed using two‐tailed *t*‐tests. Values represent the means ± SEM; *P < 0.05, **P < 0.005

Although several compounds have been identified previously from CJM, their effects on the Hif‐2α regulation of OA development remain largely unknown. Therefore, we designed an in vitro analysis to identify the suppressive effects of several individual components of CJM on Hif‐2α regulation. The contents of cirsimaritin, cirsimarin and apigenin were determined by HPLC separation of total CJM extract (Figure 4A). Although cirsimarin (Figure 4B) and cirsimaritin (Figure 4C) reduced Cox‐2 expression, they did not effectively suppress IL‐1β‐induced Hif‐2α, Mmp3 and Mmp13 up‐regulation in articular chondrocytes. However, apigenin gradually attenuated IL‐1β‐induced Hif‐2α up‐regulation, as well as that of Mmp3, Mmp13, Adamts4 and Cox‐2, in a concentration‐dependent manner (Figure 4D, Figure S2B). Apigenin also restored the accumulation of extracellular sulphated proteoglycans and inhibited aggrecan release in IL‐1β‐stimulated cartilage explants (Figure S3C and D). Furthermore, Hif‐2α‐induced Mmp3, Mmp13, Adamts4 and Cox‐2 expressions were dramatically reduced by apigenin treatment (Figure 4E, Figure S5B). These data strongly suggest that apigenin attenuates the production of Mmp3, Mmp13, Adamts4 and Cox‐2 and is a key component of CJM for reducing Hif‐2α expression in articular chondrocytes.

### 3.5 ***Apigenin directly regulates Mmp3, Mmp13, Adamts4 and Cox-2 transcriptional activity and modulates NF‐***κ***B and JNK signalling pathways for Hif‐2α regulation***

**Figure 5:**
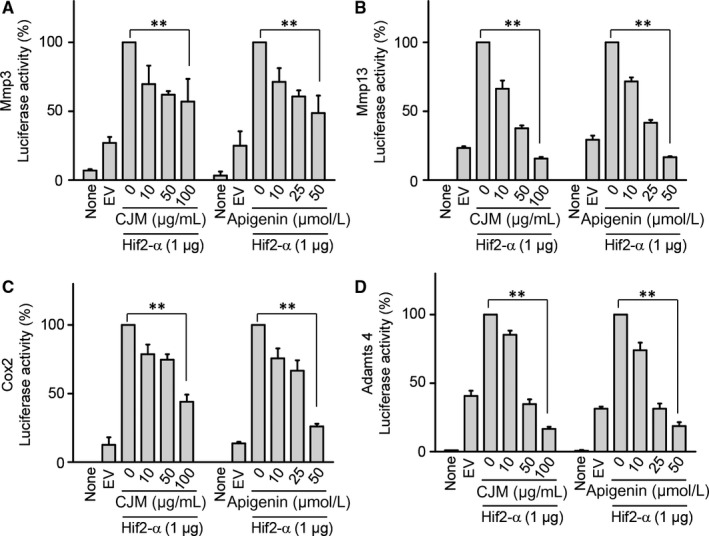
Apigenin and *Cirsium japonicum* var. *maackii* directly regulate Mmp3, Mmp13, Adamts4 and Cox‐2 transcriptional activity. A‐D, Chondrocytes were co‐transfected with Hif‐2α vector and Mmp3 (A), Mmp13 (B), Adamts4 (C) or Cox‐2 (D) promoter‐driven reporter vectors and then treated with the indicated concentrations of *Cirsium japonicum* var. *maackii* extract or apigenin for 24 h (n = 5). Data were analysed using two‐tailed *t*‐tests. Values represent the means ± SEM; **P < 0.005

**Figure 6:**
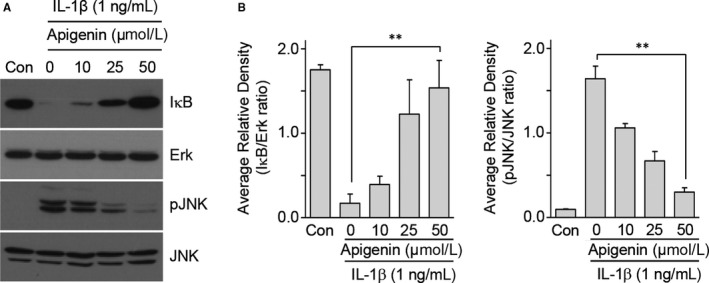
Apigenin regulates Hif‐2α expression via NF‐κB and JNK pathways. A and B, Chondrocytes were pre‐treated with different concentrations of apigenin for 24 h prior to treatment with IL‐1β (1 ng/mL) for 10 min (n = 5). IκB and phosphorylated JNK (pJNK) levels were measured by Western blotting (A) and densitometry (B). ERK and JNK were used as loading controls. Data were analysed using two‐tailed *t*‐tests. Values represent the means ± SEM; **P < 0.005

To elucidate the effect of CJM and apigenin on the direct regulation of catabolic factors by Hif‐2α, we examined Hif‐2α transactivation of the mouse promoters with or without CJM and apigenin in reporter gene assays. Transfection of a Hif‐2α expression vector in mouse articular chondrocytes elevated transcriptional activities from the promoters of Mmp3, Mmp13, Adamts4 and Cox‐2 (Figure 5A‐D). However, these elevated transcriptional activities were decreased by CJM and apigenin in a concentration‐dependent manner. These results show that CJM and apigenin affect Hif‐2α‐mediated transcription of Mmp3, Mmp13, Adamts4 and Cox‐2 mRNA. Moreover, upstream NF‐κB and JNK signalling pathways regulate Hif‐2α expression and IL‐1β, a known activator of NF‐κB and JNK signalling, increase Hif‐2α expression in chondrocytes.^14^, ^27^ Because IL‐1β‐mediated NF‐κB and JNK signalling pathways are involved in OA pathogenesis,^27^, ^28^, ^29^ we further examined whether apigenin inhibits these signalling pathways. Mouse articular chondrocytes were pre‐incubated for 24 hours either with or without apigenin and were then exposed to IL‐1β (1 ng/mL) for 10 minutes. NF‐κB and JNK signalling pathways were analysed by Western blotting. IκB degradation and JNK phosphorylation induced by IL‐1β stimulation were prevented by apigenin preincubation (Figure 6A and 6). Collectively, our results clearly indicate that Hif‐2α expression is suppressed by apigenin treatment via the inhibition of NF‐κB and JNK signalling pathways during OA development.

## 4 DISCUSSION

Osteoarthritis should not be considered a single disease; it is associated with multiple risk factors such as age, joint trauma and mechanical stress.^1^, ^2^, ^30^ In both clinical and experimental OA, cartilage destruction and inflammation are the final biological results of MMP activation and expression of ADAMTS and COX‐2 respectively.^4^, ^8^, ^11^, ^12^, ^13^ Upstream and downstream regulators of MMP and COX‐2 expression are known to be pro‐inflammatory cytokines (eg, IL‐1β, IL‐6, IL‐17 and TNF‐α) and transcription factors (eg, NF‐κB and HIF‐2α) respectively.^4^, ^5^, ^8^, ^14^, ^25^ Non‐steroidal anti‐inflammatory drugs are the most commonly prescribed medications for MMP, COX‐2 and NF‐κB regulation as well as OA treatment.^31^ However, these drugs can cause severe side effects, such as peptic ulcers, intestinal bleeding and myocardial infarction.^32^, ^33^ In this study, we demonstrated that CJM and one of its most effective constituents, apigenin, protect against Hif‐2α‐induced OA development by blocking MMP3, MMP13 and COX‐2 expressions that are up‐regulated through the JNK and NF‐κB signalling pathways and the Hif‐2α transcription factor.

CJM is a safe perennial herb that has been used as a traditional medicine. Some reports have suggested that CJM has nutraceutical importance for the treatment of inflammation, traumatic bleeding and bone loss in menopausal women.^16^, ^17^ Other ethnopharmacologic uses of CJM include its use as a treatment for hypertension, hepatitis and diuresis.^16^, ^34^ Recent pharmacological studies have indicated that CJM has tumour inhibitory, hepatoprotective and potential antidiabetic and antioxidant activities.^35^, ^36^, ^37^ However, as the effects of CJM in chondrocytes and OA development were largely unknown, in the current study, we characterized these effects under both in vitro and in vivo OA‐mimetic conditions.

IL‐1β plays a key role in joint damage through increased matrix degradation and inflammation and previous reports have suggested a role of IL‐1β in the molecular mechanism of OA development.^4^ IL‐1β constitutes the predominant pro‐inflammatory cytokine involved in the joint destruction associated with OA and it has been widely used to mimic arthritis by its application to chondrocytes.^4^, ^5^, ^8^ Moreover, other pro‐inflammatory cytokines (IL‐6, IL‐17 and TNF‐α) are known to induce MMP3 and MMP13 expression in chondrocytes and are useful for mimicking OA conditions in vitro.^5^, ^6^, ^14^ Interestingly, CJM inhibited IL‐1β‐, IL‐17‐ and TNF‐α‐induced MMP3, MMP13, ADAMTS4, ADAMTS5 and COX‐2 expression in articular chondrocytes and it may protect against OA development in vivo.

The DMM model is an important tool for studying OA pathogenesis in vivo and is a well‐accepted OA model because of its slower OA progression and similarity to human OA development.^21^ We found that CJM protected against OA development in this DMM‐induced in vivo OA model. Furthermore, we determined whether CJM and apigenin promote accumulation of sulphated proteoglycans or inhibit aggrecan release in IL‐1β‐stimulated cartilage explants. DMMB assays showed that CJM and apigenin induced the accumulation of extracellular sulphated proteoglycans and inhibited the release of aggrecan in IL‐1β‐stimulated cartilage explants.

Various transcription factors are regulated by pro‐inflammatory cytokines.^38^ Currently, Hif‐2α activation is reported to be involved in a variety of cellular signalling pathways, including those affecting inflammation, cell survival, proliferation and differentiation.^39^ Hif‐2α is particularly activated during OA development and is one of the major regulators of inflammatory cytokine production and MMP expression in OA.^40^ Moreover, IL‐1β, IL‐6, IL‐17 and TNF‐α stimulate Hif‐2α activation to contribute to joint inflammation and cartilage destruction.^6^, ^14^, ^27^ Although Hif‐2α directly binds the promoters (‐CGTG‐) of MMPs, ADAMTS and COX‐2 and is a good therapeutic target, Hif‐2α inhibitors are still lacking for OA treatment. Herein, biochemical analysis demonstrated that CJM dramatically inhibits Hif‐2α‐induced MMP3, MMP13, ADAMTS4 and COX‐2 expression as well as cartilage destruction under in vitro and in vivo OA‐mimetic conditions and that it can be used potentially as a natural Hif‐2α inhibitor.

CJM contains many medicinal components. Cirsimarin, cirsimaritin and apigenin, the biologically important flavones reported to be present in CJM, are known to process beneficial pharmacological effects. Cirsimarin has antilipolytic and antioxidant activity^19^; cirsimaritin has antibacterial, anti‐inflammatory and antioxidant properties^20^, ^21^; and apigenin has anti‐inflammatory, antiangiogenic and anticarcinogenic effects in cell culture and in various animal models.^22^ Although these compounds have specific effects on various disease conditions, the effects of cirsimarin, cirsimaritin and apigenin on OA development and mechanisms of transcription factor regulation require more study.

In the present investigation, although IL‐1β‐induced COX‐2 expression was inhibited by cirsimarin, cirsimaritin and apigenin, the expression of MMP3 and MMP13 was only blocked by apigenin treatment. Up‐regulation of Hif‐2α‐induced MMP3, MMP13, ADAMTS4 and COX‐2 expression and transcription was dramatically reduced by apigenin treatment in a concentration‐dependent manner.

Interestingly, although Ad‐Hif‐2α infection promoted exogenous Hif‐2α expression, we could detect slightly decreased Hif‐2α expression by CJM and apigenin treatment in Figures 3B and 4D. The stability of Hif‐2α is controlled by expression of Hsp90 and proteasomal degradation mediated by E3 ubiquitin ligase.^41^, ^42^ Notably, apigenin manipulates the ubiquitin‐proteasome system and reduces the expression of Hsp90 in chondrocytes.^43^, ^44^ Apigenin may regulate protein stabilization of endogenous and exogenous Hif‐2α by regulation of the ubiquitin‐proteasome degradation system and Hsp90 expression. Furthermore, Figure S5 and a previous report^6^ suggest that Hif‐2α induces IL‐6 expression and that IL‐6 can regulate Hif‐2α expression. This suggests that inhibition of Hif‐2α‐induced IL‐6 expression by CJM or apigenin treatment can reduce endogenous Hif‐2α expression. Moreover, Hif‐2α binds to its own promoter in a positive feedback loop^45^, ^46^ and the NF‐κB and JNK signalling pathways are upstream molecular mechanisms responsible for inducing Hif‐2α expression in articular chondrocytes.^14^, ^27^ Our biochemical analysis demonstrated that the underlying molecular mechanism of apigenin activity is indeed associated with Hif‐2α regulation via the JNK and NF‐κB signalling pathways. Consequently, CJM and apigenin can reduce expression levels of endogenous and exogenous Hif‐2α transcripts and protein by regulation of the ubiquitin‐proteasome degradation system, Hsp90 expression or positive feedback of Hif‐2α expression.

In summary, our results collectively indicated that the up‐regulation of MMP3, MMP13 and COX‐2 expression and cartilage destruction were inhibited by CJM via Hif‐2α regulation under in vitro and in vivo OA‐mimetic conditions. Furthermore, apigenin is an isolated compound from CJM, which effectively blocks MMP3, MMP13 and COX‐2 expression through Hif‐2α inhibition via the NF‐κB and JNK signalling pathways. Therefore, it appears that CJM and one of the most effective isolated compounds from CJM, apigenin, function as Hif‐2α inhibitors and merit consideration as OA therapeutic agents for blocking cartilage destruction and inflammation.

## CONFLICTS OF INTEREST

The authors confirm that there are no conflicts of interest.

## AUTHOR CONTRIBUTIONS

CC and SY were in charge of conception, design, analysis and interpretation of data. CC, L‐JK and DJ performed the in vivo and in vitro experiments. CSK, CHP, YSS and S‐iE contributed reagents, materials and analytical tools for the study. JJ, HL, SC, SJH, EO and JN performed reporter gene assay, DMMB and cartilage explant analysis. EP and S‐YJ performed HPLC analyses. CC and SY wrote the paper. CC and SY have full access to the data and take responsibility for the integrity and accuracy of the data analysis.

## References

[1] Feldmann M . Pathogenesis of arthritis: recent research progress. Nat Immunol. 2001;2:771‐773. 11526382 10.1038/ni0901-771

[2] Maldonado M , Nam J . The role of changes in extracellular matrix of cartilage in the presence of inflammation on the pathology of osteoarthritis. Biomed Res Int. 2013;2013:5369‐10. 10.1155/2013/284873 PMC3771246 24069595

[3] Goldring MB , Otero M . Inflammation in osteoarthritis. Curr Opin Rheumatol. 2011;23(5):471‐478. 21788902 10.1097/BOR.0b013e328349c2b1 PMC3937875

[4] Aida Y , Maeno M , Suzuki N , et al. The effect of IL‐1beta on the expression of matrix metalloproteinases and tissue inhibitors of matrix metalloproteinases in human chondrocytes. Life Sci. 2005;77(25):3210‐3221. 15979654 10.1016/j.lfs.2005.05.052

[5] Kobayashi M , Squires GR , Mousa A , et al. Role of interleukin‐1 and tumor necrosis factor alpha in matrix degradation of human osteoarthritic cartilage. Arthritis Rheum. 2005;52:128‐135. 15641080 10.1002/art.20776

[6] Ryu JH , Yang S , Shin Y , et al. Interleukin‐6 plays an essential role in hypoxia‐inducible factor 2a‐induced experimental osteoarthritis. Arthritis Rheum. 2011;63(9):2732‐2743. 21590680 10.1002/art.30451

[7] Wang M , Sampson ER , Jin H , et al. MMP13 is a critical target gene during the progression of osteoarthritis. Arthritis Res Ther. 2013;15(1):5369‐5379. 10.1186/ar4133 PMC3672752 23298463

[8] Gosset M , Pignet A , Salvat C , et al. Inhibition of matrix metalloproteinase‐3 and ‐13 synthesis induced by IL‐1 betea in chondrocytes from mice lacking microsomal prostaglandin E synthase‐1. J Immunol. 2010;185(10):6244‐6252. 20943996 10.4049/jimmunol.0903315

[9] Fosang AJ , Last K , Knäuper V , Murphy G , Neame PJ . Degradation of cartilage aggrecan by collagenase‐3 (MMP‐13). FEBS Lett. 1996;380:17‐20. 8603731 10.1016/0014-5793(95)01539-6

[10] Akhtar N , Khan NM , Ashruf OS , Haqqi TM . Inhibition of cartilage degradation and suppression of PGE2 and MMPs expression by pomegranate fruit extract in a model of posttraumatic osteoarthritis. Nutrition. 2017;33:5369‐13. 10.1016/j.nut.2016.08.004 PMC5137805 27908544

[11] Verma P , Dalal K . ADAMTS‐4 and ADAMTS‐5: key enzymes in osteoarthritis. J Cell Biochem. 2011;112(12):3507‐3514. 21815191 10.1002/jcb.23298

[12] Tchetina EV , Di Battista JA , Zukor DJ , Antoniou J , Poole AR . Prostaglandin PGE2 at very low concentrations suppresses collagen cleavage in cultured human osteoarthritic articular cartilage: this involves a decrease in expression of proinflammatory genes, collagenases and COL10A1, a gene linked to chondrocyte hypertrophy. Arthritis Res Ther. 2007;9(4):5369‐9. 10.1186/ar2273 PMC2206385 17683641

[13] Hardy MM , Seibert K , Manning PT , et al. Cyclooxygenase 2‐ dependent prostaglandin E2 modulates cartilage proteoglycan degradation in human osteoarthritis explants. Arthritis Rheum. 2002;46:1789‐1803. 12124863 10.1002/art.10356

[14] Yang S , Kim J , Ryu JH , et al. Hypoxia‐inducible factor‐2alpha is a catabolic regulator of osteoarthritic cartilage destruction. Nat Med. 2010;16(6):687‐693. 20495569 10.1038/nm.2153

[15] Ishida H , Umino T , Tsuji K , Kosuge T . Studies on antihemorrhagic substances in herb classified as hemostatics in Chinese medicine. VII on the antihemorrhagic principle in *Cirsium Japonicum* DC. Chem Pharm Bull. 1987;35:861‐864. 3594697 10.1248/cpb.35.861

[16] Noh Y‐H , Lee JW , Park J , et al. Natural substance MS‐10 improves women's health via regulation of estrogen receptor. J Korean Soc Food Sci Nutr. 2016;45:903‐910.

[17] Kim YO , Kim JS , Lee SW , Jo IH , Na SW . Posteoprotective effect of extract from *Cirsium japonicum* var. *ussuriense* in ovariectomized rats. Korean J Med Crop Sci. 2015;23:5369‐7.

[18] Rodriguez JP , Lee J , Park JY , et al. HPLC‐UV analysis of sample preparation influence on flavonoid yield from *Cirsium japonicum* var. *maackii. Appl* . Biol Chem. 2017;60(5):519‐525.

[19] Zarrouki B , Pillon NJ , Kalbacher E , et al. potent antilipogenic flavonoid, decreases fat deposition in mice intra‐abdominal adipose tissue. Int J Obes. 2010;34:1566‐1575. 10.1038/ijo.2010.85 20458325

[20] Banso A . Phytochemical and antibacterial investigation of bark extracts of *Acacia nilotica* . J Med Plants Res. 2009;3:082‐085.

[21] Kuo CF , Su JD , Chiu CH , et al. Anti‐inflammatory effects of supercritical carbon dioxide extract and its isolated carnosic acid from *Rosmarinus officinalis* leaves. J Agric Food Chem. 2011;59:3674‐3685. 21375325 10.1021/jf104837w

[22] Shukla S , Gupta S . Apigenin: a promising molecule for cancer prevention. Pharm Res. 2010;27(6):962‐978. 20306120 10.1007/s11095-010-0089-7 PMC2874462

[23] Jeon J , Kang L‐J , Lee KM , et al. 3'‐Sialyllactose protects against osteoarthritic development by facilitating cartilage homeostasis. J Cell Mol Med. 2018;22(1):57‐66. 28782172 10.1111/jcmm.13292 PMC5742729

[24] Glasson SS , Blanchet TJ , Morris E . The surgical destabilization of the medial meniscus (DMM) model of osteoarthritis in the 129/SvEv mouse. Osteoarthritis Cartilage. 2007;15:1061‐1069. 17470400 10.1016/j.joca.2007.03.006

[25] Kang L‐J , Kwon E‐S , Lee KM , et al. 3'‐Sialyllactose as an inhibitor of p65 phosphorylation ameliorates the progression of experimental rheumatoid arthritis. Br J Pharmacol. 2018;175(23):4295‐4309. 30152858 10.1111/bph.14486 PMC6240131

[26] Stanton H , Golub SB , Rogerson FM , Last K , Little CB , Fosang AJ . Investigating ADAMTS‐mediated aggrecanolysis in mouse cartilage. Nat Protoc. 2011;6(3):388‐404. 21372818 10.1038/nprot.2010.179

[27] Yang S , Ryu J‐H , Oh H , et al. NAMPT (visfatin), a direct target of hypoxia‐inducible factor‐2α, is an essential catabolic regulator of osteoarthritis. Ann Rheum Dis. 2015;74(3):595‐602. 24347567 10.1136/annrheumdis-2013-204355 PMC4345811

[28] Domagala F , Martin G , Bogdanowicz P , et al. Inhibition of interlukin‐1beta‐induced activation of MEK.ERK pathway and DNA binding of NF‐kappaB and AP‐ 1: potential mechanism for Diacerein effect in osteoarthritis. Biorheology. 2006;43:577‐587. 16912429

[29] Fan Z , Yang H , Bau B , Söder S , Aigner T . Role of mitogen‐activated protein kinases and NFκB on IL‐1β‐induced effects on collagen type II, MMP‐1 and 13 mRNA expression in normal articular human chondrocytes. Rheumatol Int. 2006;26(10):900‐903. 16468044 10.1007/s00296-006-0114-7

[30] Saklatvala J . Inflammatory signaling in cartilage: MAPK and NF‐kappaB pathways in chondrocytes and the use of inhibitors for research into pathogenesis and therapy of osteoarthritis. Curr Drug Targets. 2007;8(2):305‐313. 17305508 10.2174/138945007779940115

[31] Torzilli PA , Bhargava M , Chen CT . Mechanical loading of articular cartilage reduces IL‐1‐induced enzyme expression. Cartilage. 2011;2(4):364‐373. 22039566 10.1177/1947603511407484 PMC3203026

[32] Bacchi S , Palumbo P , Sponta A , Coppolino MF . Clinical pharmacology of non‐steroidal anti‐inflammatory drugs; a review. Antiinflamm Antiallergy Agents Med Chem. 2012;11(1):52‐64. 22934743 10.2174/187152312803476255

[33] Quan L‐D , Thiele GM , Tian J , Wang D . The development of novel therapies for rheumatoid arthritis. Expert Opin Ther Pat. 2008;18(7):723‐738. 19578469 10.1517/13543776.18.7.723 PMC2491719

[34] Rogoveanu OC , Streba CT , Vere CC , et al. Superior digestive tract side effects after prolonged treatment with NSAIDs in patients with osteoarthritis. J Med Life. 2015;8(4):458‐461. 26664470 PMC4656952

[35] Park JC , Lee JH , Choi JS . A flavone diglycoside from *Cirsium japonicum* var. ussurines. Phytochemistry. 1995;39:261‐262.

[36] Liu S , Zhang J , Li D , et al. Anticancer activity and quantitative analysis of flavone of *Cirsium Japonicum* . Nat Prod Res. 2007;21:915‐922. 17680503 10.1080/14786410701494686

[37] Yin Y , Heo SI , Wang MH . Antioxidant and anticancer activities of methanol and water extracts from leaves of *Cirsium Japonicum* . J Korean Soc Appl Biol Chem. 2008;51:160‐164.

[38] Liao Z , Wu Z , Wu M . *Cirsium Japonicum* flavones enhance adipocyte differentiation and glucose uptake in 3T3‐L1 cells. Biol Pharm Bull. 2012;35:855‐860. 22687475 10.1248/bpb.35.855

[39] Dinarello CA . Overview of the interleukin‐1 family of ligands and receptors. Semin Immunol. 2013;25(6):389‐393. 24275600 10.1016/j.smim.2013.10.001

[40] Palazon A , Goldrath AW , Nizet V , Johnson RS . HIF transcription factors, inflammation, and immunity. Immunity. 2014;41(4):518‐528. 25367569 10.1016/j.immuni.2014.09.008 PMC4346319

[41] Fujita N , Chiba K , Shapiro IM , Risbud MV . HIF‐1α and HIF‐2α degradation is differentially regulated in nucleus pulposus cells of the intervertebral disc. J Bone Miner Res. 2012;27(2):401‐412. 21987385 10.1002/jbmr.538 PMC3260409

[42] Mehmood K , Zhang H , Iqbal MK , et al. In vitro effect of apigenin and danshen in tibial dyschondroplasia through inhibition of heat‐shock protein 90 and vascular endothelial growth factor expressions in avian growth plate cells. Avian Dis. 2017;61(3):372‐377. 28957003 10.1637/11641-032817-RegR

[43] Way TD , Kao MC , Lin JK . Apigenin induces apoptosis through proteasomal degradation of HER2/*neu* in HER2/*neu*‐overexpressing breast cancer cells via the phosphatidylinositol 3‐kinase/Akt‐dependent pathway. J Biol Chem. 2004;279(6):4479‐4489. 14602723 10.1074/jbc.M305529200

[44] Singh V , Sharma V , Verma V , et al. Apigenin manipulates the ubiquitin‐proteasome system to rescue estrogen receptor‐β from degradation and induce apoptosis in prostate cancer cells. Eur J Nutr. 2015;54(8):1255‐1267. 25408199 10.1007/s00394-014-0803-z

[45] Hashimoto T , Shibasaki F . Hypoxia‐inducible factor as an angiogenic master switch. Front Pediatr. 2015;3:33. 25964891 10.3389/fped.2015.00033 PMC4408850

[46] Chen L , Endler A , Uchida K , et al. Int6/eIF3e silencing promotes functional blood vessel outgrowth and enhances wound healing by upregulating hypoxia‐induced factor 2α expression. Circulation. 2010;122(9):910‐919. 20713899 10.1161/CIRCULATIONAHA.109.931931

