# Supplementary material for: Cirsium japonicum var. maackii and apigenin block Hif‐2α‐induced osteoarthritic cartilage destruction
Source: J Cell Mol Med. 2019 May 31;23(8):5369–79. doi: 10.1111/jcmm.14418 (PMC6652892; doi:10.1111/jcmm.14418)
Supplement: Supplementary file 1 [file JCMM-23-5369-s001.pdf]

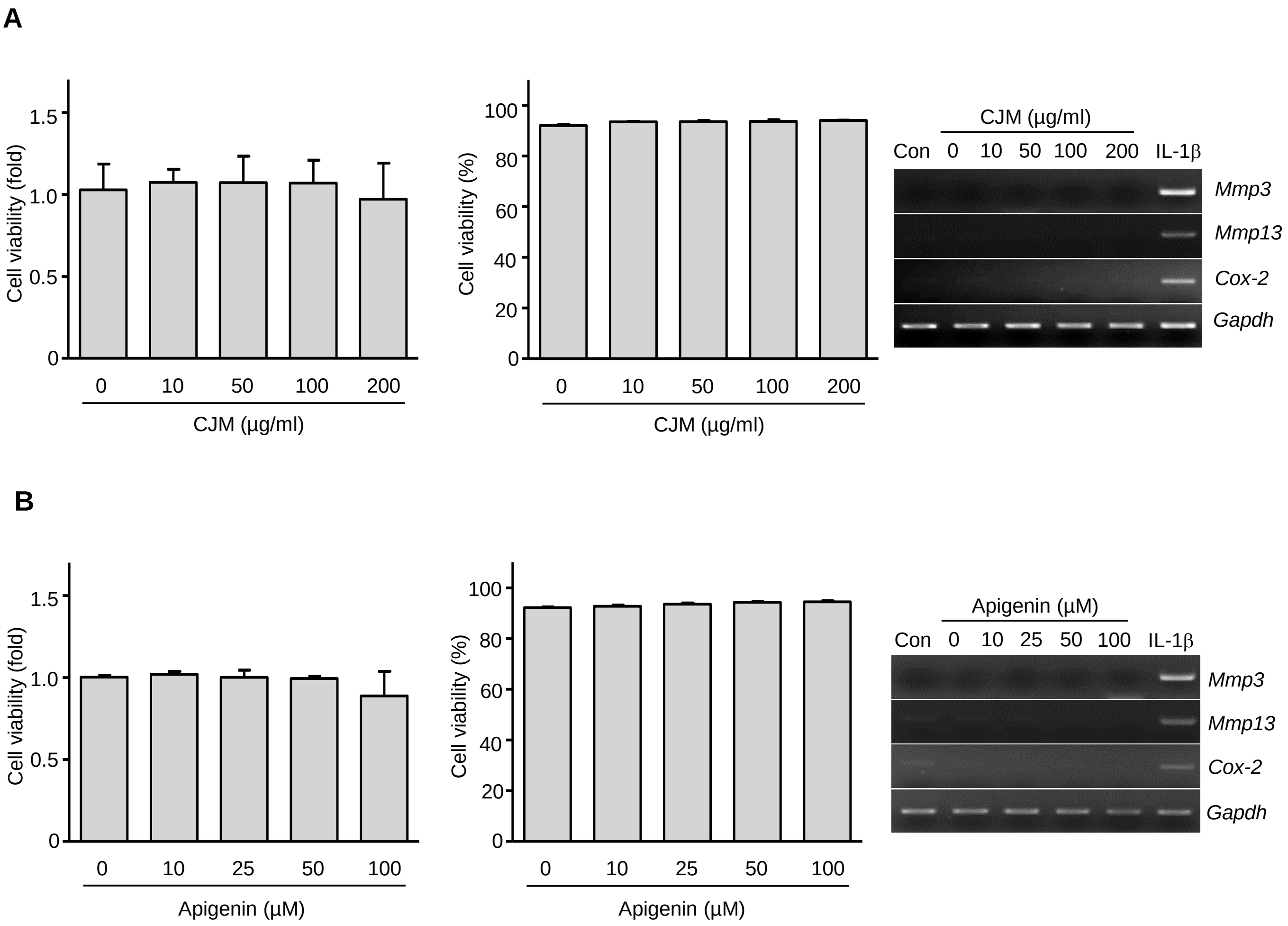

Figure S1. Effects of chondrocyte viability and cytotoxicity as detected by WST-1, LDH, and PCR assays. Effect of *Cirsium japonicum* var. *maackii* extract (**A**) and apigenin (**B**) on chondrocyte viability and cytotoxicity with WST-1 (**A, B**; left panel), LDH assay (**A, B**; middle panel) and catabolic factor expression (**A, B**; right panel). Data were analyzed using two-tailed *t*-tests. Values represent the means ± SEM. $^{**}P < 0.005$.

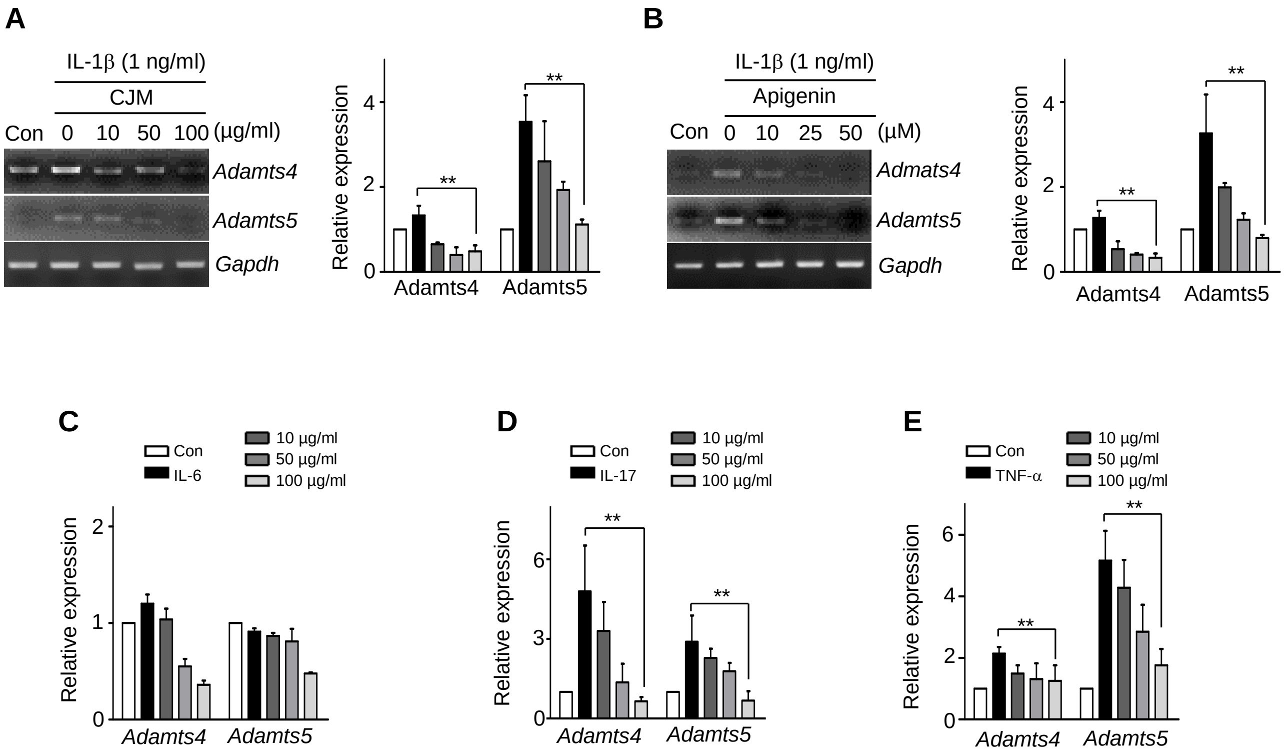

Figure S2. *Cirsium japonicum* var. *maackii* inhibits IL-1β-, IL-17-, and TNF-α-induced Adamts4 and Adamts5 expression in articular chondrocytes. (**A**, **B**) Chondrocytes treated with IL-1β (1 ng/ml) were treated with or without various concentrations of *Cirsium japonicum* var. maackii extract (**A**) and apigenin (**B**) for 24 h (n = 5). The expression of Adamts4 and Adamts5 was determined by RT-PCR (**A**, **B**; left panel) and qRT-PCR (**A**, **B**; right panel). Chondrocytes treated with IL-6 (100 ng/ml) (**C**), IL-17 (10 ng/ml) (**D**), and TNF-α (50 ng/ml) (**E**) were co-treated with *Cirsium japonicum* var. maackii extract for 24 h at the indicated concentrations. The expression of Adamts4 and Adamts5 was determined by qRT-PCR (n = 5). Data were analyzed using two-tailed t-tests. Values represent the means ± SEM (**$P < 0.005$).

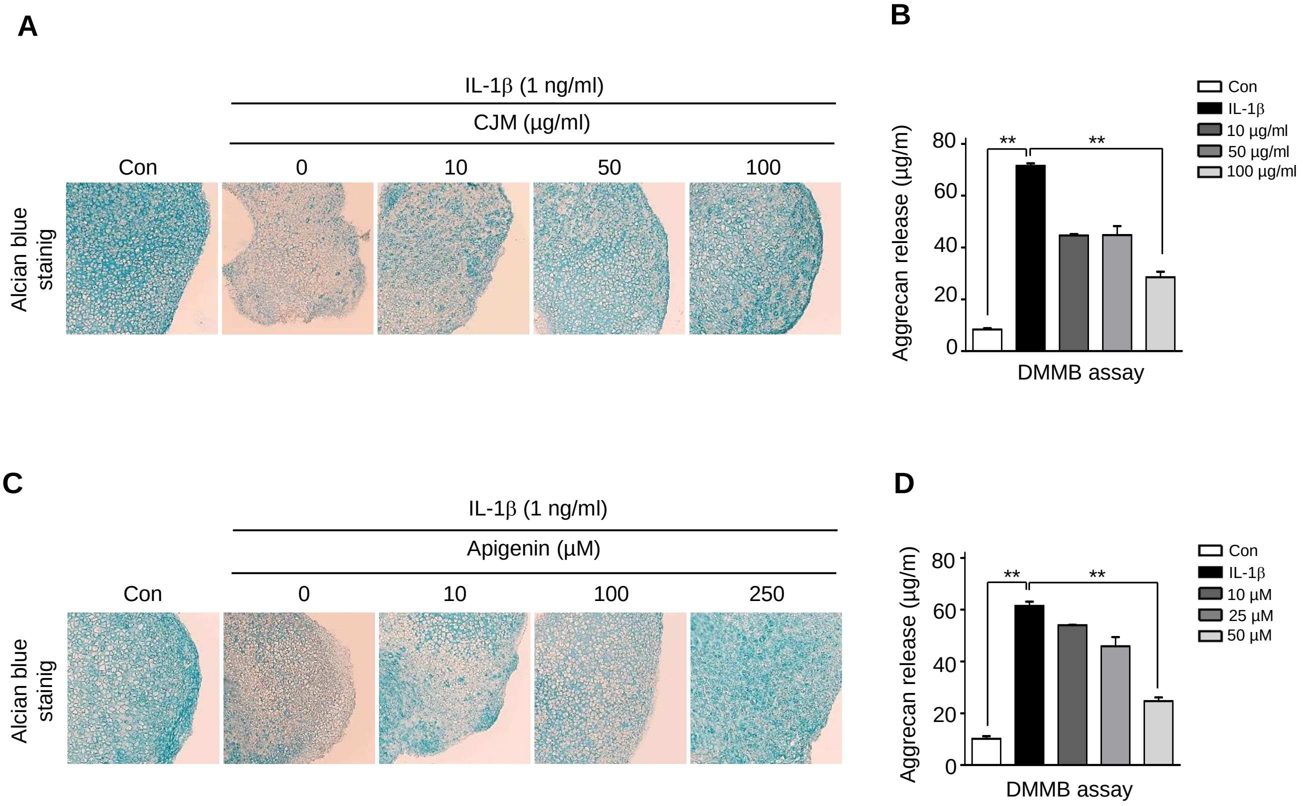

Figure S3. *Cirsium japonicum* var. *maackii* (CJM) and apigenin promote accumulation of sulfated-proteoglycans and inhibit aggrecan release. (**A, C**) Articular cartilage explants were treated with CJM extract (**A**) and apigenin (**C**) at the indicated concentrations. Following 72 h of explant culture, the accumulation of sulfated proteoglycans was assessed by Alcian blue staining. (**B**, **D**) Aggrecan release was measured in conditioned medium using a DMMB assay. Data were analyzed using one-way ANOVA with Bonferroni's test. Values represent the means ± SEM (**$P < 0.005$).

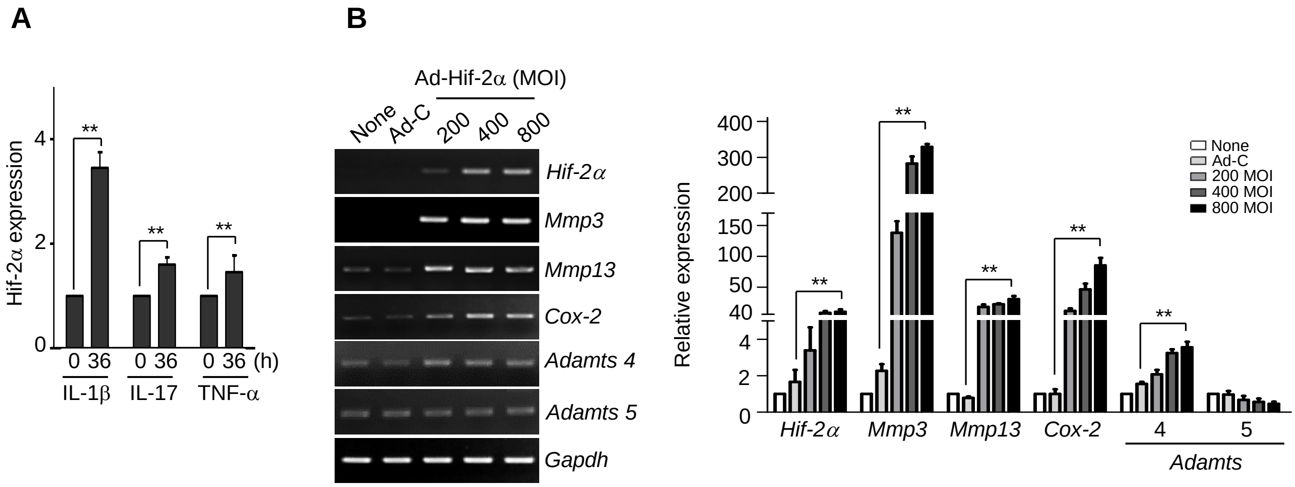

Figure S4. (**A**) qRT-PCR analyses of Hif-2α expression in articular chondrocytes treated with IL-1β, IL-17, or TNF-α. (**B**) RT-PCR (left panel) and qRT-PCR (right panel) analysis of cells transduced with or without control adenovirus (Ad-C) or the indicated amounts (in multiplicity of infection (MOI)) of Ad-Hif-2α. Data were analyzed using two-tailed *t*-tests. Values represent the means ± SEM. $^{**}P < 0.005$.

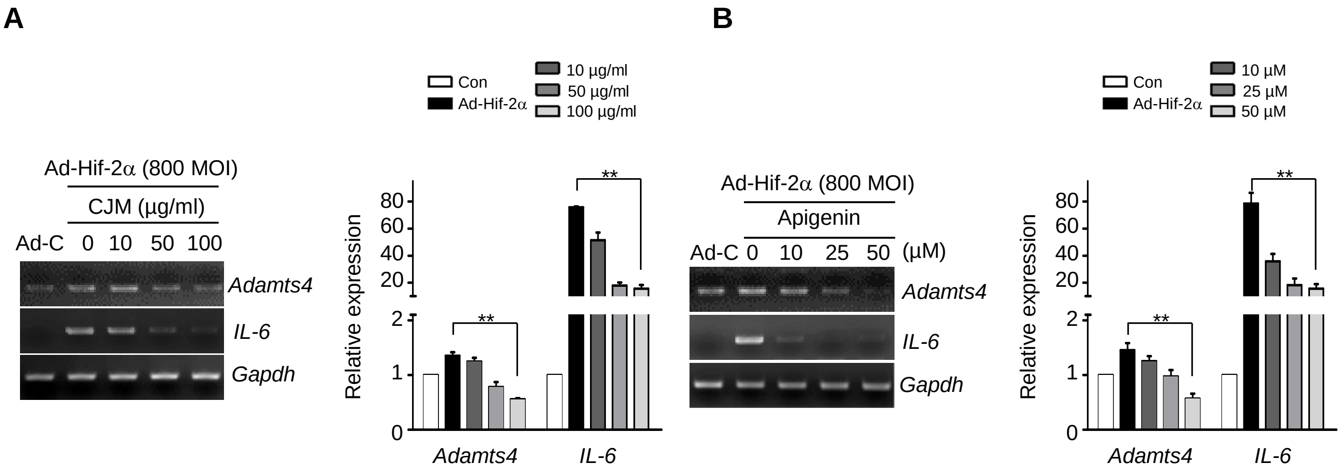

Figure S5. *Cirsium japonicum* var. *maackii* (**A**) and apigenin (**B**) inhibit Hif-2α-induced Adamts4 and IL-6 expression. (**A, B**) Chondrocytes were transduced with Ad-C or Ad-Hif-2α (800 MOI) and then treated with the indicated concentrations of *Cirsium japonicum* var. *maackii* extract (**A**) and apigenin (**B**) for 24 h. Expression of Adamts4 and IL-6 was analyzed by RT-PCR (**A, B**; left panel) and qRT-PCR (**A, B**; right panel). Data were analyzed using two-tailed *t*-tests. Values represent the means ± SEM. $^{**}P < 0.005$.
