# Supplementary material for: Cirsium japonicum var. maackii and apigenin block Hif‐2α‐induced osteoarthritic cartilage destruction
Source: J Cell Mol Med. 2019 May 31;23(8):5369–79. doi: 10.1111/jcmm.14418 (PMC6652892; doi:10.1111/jcmm.14418)
Supplement: Supplementary file 2 [file JCMM-23-5369-s002.docx]

**Supporting information**

Additional supporting information may be found in the online version of this article:

**Table S1**. Primer sequences and PCR conditions

| Gene | Origin | Strand | Sequence | Size  (bp) | AT^a^  (°C) |
| --- | --- | --- | --- | --- | --- |
| *Mmp3* | Mouse | ^b^S  ^c^As | 5'-CTGTGTGTGGTTGTGTGCTCATCCTAC-3'  5'-GGCAAATCCGGTGTATAATTCACAATC-3' | 350 | 58 |
| *Mmp13* | Mouse | S  As | 5'-TGATGGACCTTCTGGTCTTCTGGC-3'  5'-CATCCACATGGTTGGGAAGTTCTG-3' | 473 | 58 |
| *Cox-2* | Mouse | S  As | 5'-GGTCTGGTGCCTGGTCTGATGAT-3'  5'-GTCCTTTCAAGGAGAATGGTGC-3' | 724 | 65 |
| *Hif-2α* | Mouse | S  As | 5'-TCACTGCCACCCAGAAGAC-3'  5'-TGTAGGCCATGAGGTCCAC-3' | 450 | 55 |
| *Adamts4* | Mouse | S  As | 5'-TCACTGCCACCCAGAAGAC-3'  5'-TGTAGGCCATGAGGTCCAC-3' | 450 | 55 |
| *Adamts5* | Mouse | S  As | 5'-TCACTGCCACCCAGAAGAC-3'  5'-TGTAGGCCATGAGGTCCAC-3' | 450 | 55 |
| *Gapdh* | Mouse | S  As | 5'-TCACTGCCACCCAGAAGAC-3'  5'-TGTAGGCCATGAGGTCCAC-3' | 450 | 55 |

^a^AT, annealing temperature; ^b^S, sense; ^c^As, antisense
